# Lived Experience Performance to Reduce Stigma, Enhance Understanding of Gambling Harm and Change Attitudes and Behaviours of Professionals and Community Members

**DOI:** 10.1007/s10899-023-10223-0

**Published:** 2023-06-06

**Authors:** Anna C. Thomas, Hannah Portogallo, Fiona Read, Judy Avisar, Stephanie S. Merkouris, Nicki A. Dowling

**Affiliations:** 1 School of Psychology, Faculty of Health, Deakin University, Geelong, Australia; 2 Link Health and Community, Glen Waverley, VIC Australia; 3 Access Health and Community, Hawthorn, VIC Australia; 4 Self Help Addiction Resource Centre (SHARC), Carnegie, Australia

**Keywords:** Gambling, Lived experience, Affected others, Qualitative, Drama, Performance, Theatre, Behaviour change

## Abstract

Gamblers and their family members or friends (affected others) can experience stigma and shame due to gambling which can result in a reluctance to seek timely support. However, gamblers and affected others access intersecting health services and talk to friends or family, thereby providing opportunities for early intervention. *Three sides of the coin* is a group of storytellers with lived experience of gambling harm who use dramatic performance to share personal stories to enhance the understanding of gambling-related harm in allied professions and the broader community. They do this to encourage attitude and behaviour change so that gamblers and affected others receive empathy and support during encounters with these groups. A mixed-methods study was used to explore whether these performances were successful in increasing understanding and changing attitudes and behaviour of allied professionals and the community in the short and longer-term. Data collected immediately post-performance revealed that performances increased understanding of gambling, and improved attitudes and behavioural intent of audience members in relation to gamblers and affected others. Professionals also reported an increased willingness and confidence to discuss gambling harm with clients. Follow-up data demonstrated potential longer-term impact, with respondents continuing to report more positive attitudes towards those affected by gambling harm and professionals being confident to explore gambling issues in their clients and provide appropriate referrals. These finding demonstrate that performance based on lived experience can be a powerful education tool, encouraging deep connection to the issue, resulting in a nuanced understanding and sustained attitudinal and behavioural change.

## Introduction

A public health perspective considers the complexities of gambling within our communities by acknowledging the spectrum of gambling harm that impacts a significant number of people, including people who gamble, affected-others (e.g., partners, children, other family members, friends, colleagues, employers) and the broader community (Browne et al., 2016; Productivity Commission, 2010). This gambling harm can be defined as “any initial or exacerbated adverse consequence due to an engagement with gambling that leads to a decrement to the health or wellbeing of an individual, family unit, community or population” (p. 36, Browne et al.). These decrements to health and wellbeing (financial, relationship, emotional wellbeing, physical health, social and cultural capital, work/study performance) can create short-term general harm and crises, through to longer-term legacy and intergenerational impacts (Browne et al.). Gambling harm affects a significant proportion of the community. National Australian estimates indicate that approximately 9.1% of Australian adults report gambling harm to themselves and that 6.0% report being harmed by another person’s gambling (Hing et al., 2021a, 2021b).

Family and friends are therefore also harmed by gambling and, as with other addictions, they are a potential doorway to support and recovery for those experiencing addiction. This is important as shame and stigma can discourage those harmed by gambling from seeking support or treatment until a crisis point is reached (Suurvali et al., 2009). However, affected others need to know how to engage in these hard conversations and how to look after themselves. Whilst they may be the first to see or hear that gambling has become problematic, they often do not recognise what is happening or may not know how to respond appropriately (Bond et al., 2015).

Allied health services are another potential doorway to support and recovery. People affected by gambling may access a range of other allied health services, due to the consequences of gambling or in relation to other comorbid conditions including mental health, financial issues, alcohol and drug use, involvement with the justice system, and intimate partner violence (Browne et al., 2016; Cowlishaw et al., 2013; Dowling et al., 2014, 2017; Hing et al., 2021a, 2021b). In the spirit of the ‘no wrong door’ approach, this presents an opportunity for screening and early intervention in relation to gambling-related harm. This, however, will only occur where allied service workers feel confident and capable to identify and manage gambling harms in their client base (Manning et al., 2020; Rodda et al., 2018).

### Arts as a Mechanism for Change

Engagement in the arts can benefit mental, social and physical health wellbeing as well as the social determinants of health (e.g. economy, employment) (Davies et al., 2016). This includes engagement in arts as active involvement or in receipt of art such as an audience member. The arts can also be powerful tool to create change through increasing understanding and challenging preconceptions in audiences to shift attitudes and behaviour (Dunst, 2014; Fraser & Sayah, 2011; Mubangizi et al., 2022).

Forum Theatre, for example, is a participatory drama technique based on Augusto Boal’s ‘Theatre of the Oppressed’ (Sappa & Barabasch, 2019). This is an immersive method in which participant-storytellers can elicit emotional and cognitive (critical reflection) responses from the audience, confront realistic problems and—by overcoming the distinction between actor and audience—invite audiences to participate and consider their role to act (Sappa & Barabasch). The performance allows individual participant-storytellers to assume different positions on a problem and test it on stage (a ‘rehearsal for reality’), thus guiding active coping and personal control over a stressor and supporting resilience. Finally, by creating emotional dissonance, the ‘spect-actors’ are stimulated to take action (Sappa & Barabasch).

Performance arts, both live and recorded, have been used as a mechanism to change attitudes and behaviour in community members and professionals across various, health related fields (e.g., Crisp & Taket, 2022; Fraser & Sayah, 2011; Quek et al., 2012). However, there is limited contemporary literature about the use of theatre for primary, secondary or tertiary prevention of gambling harm. One example comes from Shu (2018, 2020), who writes of forum theatre and ethno-theatre approaches used in Hong Kong, where those with lived experience of gambling harm (as gamblers or affected others) share their personal stories. These studies explored the power of drama and performance arts on both the participants and the audience and supported the construct of empowerment at the personal, inter-personal and collective or societal levels. This included examining the identities formed when someone is empowered to share their personal story to advocate for change and of the audience who learn about gambling, develop confidence and the need to be aware of gambling harm in society (Shu).

### Three Sides of the Coin

Three Sides of the Coin (3SOC) empowers people with lived experience of gambling harm (3SOC storytellers) to creatively explore their own personal stories in workshops and share these through live performance. As such, they combine the power of lived experience and the language of dramatic performance. This work is led by Artistic Director Catherine Simmonds, who has been devising performances with diverse communities, since 1992. “Empathy for her is about creating a space in which to respect difference—both in workshop and on stage” (p. 189, Cummings, 2016). Through her creative workshop methods, she has rendered hundreds of stories into performance, enabling each participant to become the protagonist of their own story, exploring tensions between agency and vulnerability. She thereby creates a space to build relationships over time, to ask questions and to enable audiences to listen to the answers. Mid-career, Simmonds practice was further influenced by the Forum Theatre approach (Cummings). This recognises that a story is more than its narrative content; it is a contribution to (or intervention into) the wider process of making meaning.

There is a two-fold purpose to 3SOC and their performances. First, they provide a means for people to explore and articulate aspects of their own recovery through story, improvisation and the embodying of their own experiences. Much of the work is done as a group thereby providing the benefit of mutual support to members. Second, performances are intentionally devised, curated and delivered to increase audience understanding of gambling harm and, through this, inspire changes to attitudes and behaviour. In particular, performances aim to increase empathy, reduce stigma and shame towards people with gambling-related harm, and increase confidence to discuss gambling harm.

Following a performance, audiences are invited to actively participate by reflecting upon their feelings through a one-word response and to ask questions of the storytellers, providing for an interactive discussion led by the audience. Performances are most commonly offered as professional and sector development to staff working in non-gambling fields that intersect with gambling harm (e.g., the justice system, banking and finance, and allied health), but may also be provided to professionals working with gambling harm and the broader community.

To understand the impact of 3SOC on the 3SOC storytellers and audience members, a research project was undertaken consisting of three, linked phases: Study 1: Audience post-performance survey examining the immediate impact of the performance on audience-members’ understanding and attitudes towards gambling and gamblers; Study 2: Audience follow-up survey examining the longer term impact of the performance on audience-members’ understanding, attitudes towards gambling and gamblers, and behaviour; Study 3: Semi-structured interviews with 3SOC Storytellers exploring the impact of participation on themselves.

This paper presents the results of the first two studies focussing on the impact of performances on the audience. It aims to explore whether exposure to a 3SOC performance increases understanding of gambling and gambling harm and if it results in positive changes to attitudes and behaviours of professionals and community members in the short and longer term.

## Study 1: Post-Performance Survey

### Methods

The overall project used an action research approach (Heller, 2004) with the research examining the ability of 3SOC to effect attitudinal and behavioural change within professionals and the community via dramatic performances from people with lived experience of gambling harm. In line with the approach of 3SOC, who use principles from Forum Theatre, the audience is invited to actively participate through post-performance discussions and to reflect critically on their own attitudes and behaviours as a mechanism for creating meaningful attitudinal and behaviour change (Sappa & Barabasch, 2019). The two time points for data collection allowed the research to explore the effects of the performance and critical reflections in the short and longer term. Staff and members of 3SOC, representing lived experience of gambling harm, were also actively involved throughout the project. Study 1 aimed to examine the short-term impact of the 3SOC performance in terms of self-reported improvements in general knowledge and attitudes about gambling and in practice intentions. A mixed methods research design was used to collect quantitative and qualitative data regarding the impact of 3SOC performances on audience members immediately following a performance.

#### Participants

The audiences attending 3SOC included community members and people attending in their professional capacity (herein referred to as professionals). This included ‘allied professionals’ from sectors that intersected with gambling including mental health, financial counselling, alcohol and other drug (AOD), family violence, justice, legal, health, banking and finance sectors. At times audiences also included gambling harm specialists (herein referred to as gambling help professionals).

A total of 301 audience members (64 community members, 231 professionals, 6 unreported) attending 41 3SOC performances completed the post-performance survey over a 22-month period from April 2017 to February 2019.

The first sub-sample of attendees were 64 community members. The second sub-sample of attendees were 231 professionals who attended the performance for their professional development. These professionals were mostly employed in the mental health (n = 67), AOD (n = 23), family violence (n = 15), justice/legal (n = 12), health (n = 11) and gambling harm (n = 7) sectors. The remaining professionals were employed in other sectors or indicated multiple sectors (n = 95); or did not specify the sector in which they were employed (n = 1).

Basic demographic characteristics of the overall sample, broken down by the two subsamples are displayed in Table 1. Of the total sample, most were female and aged between 25 and 34 years of age.

**Table 1:** Study 1 sample descriptive statistics

Demographic characteristic	Community members	Professionals	Total sample^a^
Gender (female)	41 (66.1%)	162 (71.1%)	209 (70.6%)
*Age*			
18–24 years	12 (18.8%)	17 (7.5%)	29 (9.8%)
25–34 years	15 (23.4%)	74 (32.7%)	90 (30.4%)
35–44 years	5 (7.8%)	45 (19.9%)	52 (17.6%)
45–54 years	12 (18.8%)	43 (19.0%)	55 (18.5%)
55–64 years	8 (12.5%)	39 (17.3%)	47 (15.9%)
65 +	12 (18.8%)	8 (3.5%)	12 (4.1%)

Proportions vary due to small amounts of missing data

^a^Six participants did not specify the subsample to which they belonged

#### Measures

The post-performance survey included demographic items (gender, age, professional setting) and a mixture of bespoke closed and open-ended items designed to examine the experience of the performance and measure changes in attitudes and behavioural intention. Four of the five closed questions evaluated attendees’ understanding about gambling harm, empathy towards people affected by gambling harm, willingness to discuss gambling harm and confidence to ask clients about their gambling (professionals only) on a 5-point Likert-type scale: (1) strongly decreased; (2) decreased; (3) neither increased nor decreased; (4) increased; (5) strongly increased. The remaining closed question assessed opinions on whether this type of performance was a powerful way of connecting to the issue of gambling harm, which was rated on a 5-point Likert scale: (1) strongly disagree; (2) disagree; (3) neither agree nor disagree; (4) agree; (5) strongly agree.

Two open-ended questions were used to generate qualitative data in terms of changes in understanding (learnings) and attitudes towards gambling harm in audience members. A third open-ended question asked professional audience members about how their practice may change as a result of the session (behavioural intention). A final item asked participants if they were willing to be invited to participate in a follow-up survey (Study 2). Participants expressed their interest in participating in further research by selecting a check box and providing their email address.

#### Procedure

The project was approved by the Deakin University Human Research Ethics Committee (DUHREC, HEAG-191-2018). Audience members were invited to complete a short paper-based survey immediately following a 3SOC performance. Participation was voluntary and participants demonstrated their consent by returning their completed survey research team. The survey took no longer than five minutes to complete and no reimbursements or incentives were provided.

#### Data Analysis

Descriptive statistics were used to present the quantitative data, with count and percentages presented for all variables. Professionals intersecting with gambling harm (allied professionals) are the primary target audience for change for 3SOC. In contrast, gambling help professionals would be expected to have received training regarding causes and consequences of gambling harm. As such, the small group of professionals in the gambling harm sector (n = 7) were excluded from the quantitative analyses to avoid confounding results. Also excluding the one professional who did not indicate the sector in which they were employed, this left a total sample of 287 participants (64 community members, 223 allied professionals). Differences between these allied professionals and community members on each item were examined using chi-square tests for independence. For the purpose of these analyses, the response options for each item were dichotomised, with negative and neutral response options combined and compared to positive response options.

Responses to the three open ended questions were analysed using reflexive thematic analysis (Braun & Clarke, 2006, 2019). One researcher (HP) conducted the initial data analysis, reading and then re-reading responses to familiarise herself with the data then coding the data and generating a list of initial themes. The data, codes and themes were then reviewed by another researcher (AT). HP and AT then worked together to review and develop the final list of themes (Braun & Clarke). All qualitative data was analysed but particular emphasis was placed on data from allied professionals as the majority of responses came from this group and they were the primary audience in terms of desired change for 3SOC. Data for community members and gambling help professionals were also analysed and reported, but discussion is predominantly focused on allied professionals.

### Quantitative Results

Table 2 displays the post-performance survey results, broken down by the two subsamples: community members and allied professionals. The findings revealed that almost all of the sample (99%) agreed that performance by people who have experienced gambling harm was a powerful way of connecting to the issue.

**Table 2:** Post-performance survey results

Survey item	Community members	Allied professionals^a^	χ^2^	*p*-value	Combined sample
*Power of performance*^*g*^					
Performance by people who have experienced gambling harm was a powerful way of connecting to the issue^b^	60 (98.4%)	215 (98.6%)	0.023	0.879	275 (98.6%)
*General knowledge and attitudes about gambling harm *^*h*^					
Understanding about gambling harm^c^	58 (92.1%)	197 (89.5%)	0.348	0.555	255 (90.1%)
Empathy towards people affected by gambling harm^d^	60 (95.2%)	209 (95.4%)	0.004	0.948	269 (95.4%)
*Professional attitudes and practice *^*h*^					
Willingness to discuss gambling harm^e^	–	197 (90.8%)	–	–	–
Confidence in asking clients about their gambling^f^	–	187 (87.8%)	–	–	–

^a^Excluding professionals who did not indicate the sector in which they were employed (n = 1) or professionals in the gambling harm sector (gambling-help professionals) (n = 7)

^b^n = 279 (61 community members, 218 allied professionals)

^c^n = 283 (63 community members; 220 allied professionals)

^d^n = 282; (63 community members, 219 allied professionals)

^e^Administered only to allied professionals (n = 217)

^f^Administered only to allied professionals (n = 213)

^g^Proportion of sample indicating agree/strongly agree; ^h^ Proportion of sample endorsing increased/strongly increased

The majority of participants reported their understanding about gambling harm (90%) and empathy towards people affected by gambling harm (95%) had increased after watching a 3SOC performance. There were no significant differences between the two subsamples on their responses to these items. Moreover, the majority of allied professionals reported that their willingness to discuss gambling harm (91%) and their confidence to ask clients about their gambling (88%) had increased after watching the performance.

### Qualitative Results

A total of 257 individuals responded to least one of the open-ended items, the vast majority of whom were allied professionals (n = 188), with an additional 58 community members, six gambling help professionals and five others also responding. The analysis identified three themes. The first theme identified related to changes in audience-members’ understanding of gambling harm.

#### Understanding Gambling Harm

The vast majority of respondents reported an increased and more nuanced understanding of gambling harm following the performance, including in relation to the *size and scope* of the problem, the *complexity of causes and consequences* of gambling addiction and harm and the *stigma and shame* that can be experienced by both gamblers and affected others*.*

Some were surprised and even humbled to learn about the rate of gambling in Australia and the sheer size and scope of gambling losses, “Terrifying statistics for our Australian context. I’ve never heard first-hand experience of such depth around gambling. A very humble[sic] experience to listen” (allied professional).

One of the most common learnings was that gambling is complex and multi-layered, both in terms of its causes and its consequences. Respondents realised that anyone can be at risk, regardless of their upbringing, life circumstances, background or exposure, and that no one circumstance was a precursor for gambling addiction: “Anyone can become addicted” (allied professional). Instead, they discovered that the reason an individual gambles is complicated and can be subtle or pervasive in nature. Some participants highlighted that they are now more aware of precipitating factors and just how varying the experience might be for different individuals: “The endless cycle of issues that link with gambling and how gambling brings the issues” (allied professional).

Many emphasised the power of the performance in portraying the consequences of gambling for the gambler, their loved ones, and the broader community: “The performance gave me an insight into the pull of gambling once it becomes problematic and the impact this has on the individual and those who love them” (allied professional). Allied professionals emphasised increased understanding of both financial and emotional impacts: “How much money is wasted on gambling and the massive effect it has on our community” (allied professional); “I learnt about loss, grief and trauma impacts of gambling behaviour” (allied professional). Both community members and professionals discussed an increased awareness of just how vulnerable, lonely and frightened gamblers often feel: “An increased understanding about how frightening feeling compelled to gamble would feel” (allied professional).

The stigma and shame affecting both the gambler and affected others was an important sub-theme identified by both community members and allied professionals, with both groups saying they better recognised and understood the level of stigma and shame associated with gambling, which they now viewed as equivalent or worse than that associated with alcohol and drug use: “More about the stigma against gambling vs other addictions” (community member). Importantly, professionals understood the impact that stigmatisation by their sector could have, including a reluctance to disclose problems “Shame can keep people silent” (allied professional) and self-destructive thoughts “Made a sharper connection between gambling—shame—suicidality” (allied professional).

These responses demonstrate an increased understanding about the causes and complexity of gambling addiction in 3SOC audiences, as well as the scope and extent of gambling-related harm. This increased understanding led to more positive attitudes and an increased confidence and willingness to act—to engage with the problem and support those harmed by gambling. There were two aspects to these changes, discussed in the final two themes of Study 1: actively identifying gambling issues and harms, and being open to exploring gambling issues and providing support to those harmed.

#### Acting to Identify Gambling Issues and Harm

There were three elements to this theme, with respondents indicating they would be more likely to *look for and talk about gambling* and to respond to *signs of gambling issues* as well as having an increased confidence in *knowing what to say* and what not to say.

Both professionals and community members expressed more willingness to look for and talk about gambling: “More open to conversations about it with others” (community member). Importantly, there was strong evidence that professionals were now more willing to ask the question: “Be more direct in asking about gambling. Help people see the bigger picture” (allied professional). This change in behavioural intention appeared to derive from a greater understanding of the issue and being more aware that people with gambling-related harm will appreciate and respond to questions, as well as the potential for change.

Most allied professionals also explained that, as well as directly asking about gambling, they would be more likely to respond to signs of a gambling issue. “If I notice issues with money, I’ll be much more likely to ask if gambling is a problem for the person I’m talking to” (allied professional). This change was also evident in community members: “Be alert for it – speak up if I have a friend or family member I suspect is going through this” (community member).

Importantly, participants also reported that the performance taught them how to respectfully approach people with gambling-related harm, including what is, and is not, appropriate to say: “How to approach someone about gambling harm and help them speak up. What not to say” (allied professional).

#### Exploring the Issue and Supporting those Harmed by Gambling

An important theme among allied professionals was an increased openness to exploring gambling harm within their practice and supporting clients affected by gambling. This was demonstrated in a variety of ways and reflected a significant and positive shift in attitude and behavioural intention of these professionals.

The increased understanding of the causes of gambling issues and associated stigma and shame led professionals to question prior assumptions: “I think it will change my thinking about gambling i.e., “I don’t get it”, “waste of money”, to more understanding of the addiction” (allied professional).

Allied professionals articulated their intention to work in a respectful and empathic manner with clients experiencing gambling issues and harm: “Be more empathic towards those with gambling concerns. Non-judgemental listening” (allied professional) and try to reduce shame and stigma that clients may feel: “I will be more conscious of asking people/clients about their gambling behaviour, I’ll be more aware of the shame and stigma surrounding the behaviour” (allied professional).

Allied professionals were more open to actively talk about and integrate gambling-related issues within their practice: “More open to understanding/ being sensitively curious to open a conversation” (allied professional); being sensitive to the bigger picture: “Seeing it more in combination with other life stressors. More understanding” (profession not stated); and considering the impact of gambling on clients: “As a professional, I might consider why they gamble and their mental state” (allied professional).

Finally, in addition to integrating gambling support into their own practice, allied professionals were clear about the need to support clients to find additional services where necessary: “If gambling is highlighted as an issue of the client, supporting them to link in with appropriate services” (allied professional), recognising that this may not be easy for those affected by gambling to access: “Assistance for non-English speaking victims can be hard to access due to language barriers and stereotypes” (allied professional).

## Study 2: Follow-up Survey

### Methods

This study aimed to examine the longer-term impact of the 3SOC performance in terms of self-reported improvements in general knowledge and attitudes about gambling harm and in professional attitudes and practice. A mixed methods research design was again used to collect quantitative and qualitative data regarding the longer-term impact of performances by 3SOC on audience members, with data collected in the months following the initial performance. Data was collected over a 6-month period between March and August 2019.

#### Participants

A total of 116 audience members (26 community members, 83 professionals, 7 unreported) who had attended 3SOC performances in the past took part in the follow-up survey.

The first sub-sample of attendees were 26 community members, most of whom were employed full-time (n = 7), employed part-time or causal (n = 7) or retired (n = 5). The second sub-sample of attendees were 83 professionals, who were employed in the mental health (n = 20), gambling harm (n = 17), health (n = 9), AOD (n = 4), family violence (n = 1), and justice/legal (n = 1) sectors; the remaining professionals were employed in other sectors (n = 30) or did not specify the sector in which they were employed (n = 1).

Of the professionals, 66 allied professionals were community workers (n = 10), social workers (n = 9), mental health workers (n = 7), financial counsellors (n = 4), counsellors (n = 5), occupational therapists (n = 2), AOD counsellors (n = 2), psychologists (n = 1), and family violence workers (n = 1); the remaining allied professionals were employed in other professions (n = 21) or did not specify the profession in which they were employed (n = 4). Allied professionals reported they worked in community mental health services (n = 20), community outreach settings (n = 5), AOD services (n = 3), outpatient hospital settings (n = 2), acute or inpatient hospital settings (n = 1), private practice (n = 1), and/or university clinics (n = 1); the remaining allied professionals were employed in other settings (n = 35), with some reporting working in multiple settings. These allied professionals reported they had worked professionally for 1 to 44 years (M = 14.6, SD = 10.3, median = 14.0).

In contrast, gambling help professionals worked in community education (n = 7), Gamblers Help face-to-face services (n = 3), financial counselling (n = 2); the remaining gambling help professionals did not specify the type of service they were employed in (n = 4) or did not specify the service in which they were employed (n = 1). These gambling help professionals reported they had worked professionally for 1–45 years (M = 16.8, SD = 13.7, median = 17).

Basic demographic characteristics of the overall sample, broken down by the two subsamples are displayed in Table 3. Of the total sample, most were female, aged between 35 and 44 years of age, had a postgraduate qualification, were born in Australia and were in a couple with children still living at home.

**Table 3:** Study 2 sample descriptive statistics

Demographic characteristic	Community members	Professionals	Total sample^a^
Gender (female)	21 (80.8%)	63 (75.9%)	84 (77.1%)
*Age*			
18–24	2 (7.7%)	2 (2.4%)	4 (3.7%)
25–34	3 (11.5%)	14 (16.9%)	17 (15.6%)
35–44	2 (7.7%)	23 (27.7%)	25 (22.9%)
45–54	5 (19.2%)	18 (21.7%)	23 (21.1%)
55–64	7 (26.9%)	23 (27.7%)	30 (27.5%)
65 +	7 (26.9%)	3 (3.6%)	10 (9.2%)
*Education level*			
Did not complete high school	0 (0%)	1 (1.3%)	1 (1.0%)
Completed high school	0 (0%)	1 (1.3%)	1 (1.0%)
Vocational or Trade qualification	4 (16.7%)	10 (12.8%)	14 (13.7%)
Undergraduate degree	12 (50.0%)	23 (29.5%)	35 (34.3%)
Postgraduate qualification	8 (30.8%)	42 (50.6%)	50 (49.0%)
Other	0 (0%)	1 (1.3%)	1 (1.0%)
Country of birth (Australia)	15 (62.5%)	59 (75.6%)	74 (72.5%)
*Household composition*			
Couple with no children	4 (16.7%)	12 (15.4%)	16 (15.7%)
Couple with children still at home	8 (33.3%)	27 (34.6%)	35 (34.3%)
Couple with children not living at home	4 (16.7%)	16 (20.5%)	20 (19.6%)
Single person household (no children)	3 (12.5%)	9 (11.5%)	12 (11.8%)
Single with children not living at home	2 (8.3%)	2 (2.6%)	4 (3.9%)
Group or shared household	2 (8.3%)	5 (6.4%)	7 (6.9%)
In some other arrangement	1 (4.2%)	2 (2.6%)	3 (2.9%)

Proportions vary due to small amounts of missing data

^a^Seven participants did not specify the subsample to which they belonged

#### Measures

The survey included demographic items (e.g., gender, age, professional group, education level, country of birth, household composition, employment status [community members only], employment sector [professionals only], profession [professionals only], workplace setting [professionals only] and years of working professionally [professionals only]) and a mixture of bespoke open and closed items designed to examine participants’ experience of the performance and measure changes in attitudes and behavioural intention.

Two closed questions evaluated attendees’ views on the power of the performance (e.g., the use of theatre was a powerful way to present the material) on a 5-point Likert-type scale: (1) strongly disagree; (2) disagree; (3) neither agree nor disagree; (4) agree; (5) strongly agree. Two closed questions evaluated changes in general knowledge and gambling harm since the performance (e.g., understanding about gambling harm) on 5-point Likert-type scale: (1) strongly decreased; (2) decreased; (3) neither increased nor decreased; (4) increased; (5) strongly increased. Four closed questions evaluated attendees’ current general knowledge and attitudes about gambling harm (e.g., empathy towards people who have experienced gambling harm) on a 5-point Likert-type scale: (1) strongly disagree; (2) disagree; (3) neither agree nor disagree; (4) agree; (5) strongly agree.

Four closed items administered to professionals only evaluated changes in professional attitudes since the performance (e.g., willing to discuss gambling harm) on 5-point Likert-type scale: (1) strongly decreased; (2) decreased; (3) neither increased nor decreased; (4) increased; (5) strongly increased. Five closed questions administered to professionals only evaluated their current professional attitudes and practice (e.g., confidence in asking clients about their gambling) on a 5-point Likert-type scale: (1) strongly disagree; (2) disagree; (3) neither agree nor disagree; (4) agree; (5) strongly agree. Professionals were also asked about how often they informally asked their clients about their gambling on a 5-point Likert type scale: (1) never; (2) rarely; (3) sometimes; (4) often; (5) almost always. Professionals were also asked if their organisation has a gambling screen in its client intake tool: (1) No; (2) Yes; (3) Don’t know.

Three open-ended items were used to generate qualitative data relating to: (1) imagery, words or feelings associated with the performance; (2) changes in attitude towards gambling harm as a result of the performance; and (3) changes in practice as a result of the performance (asked of professionals only).

#### Procedure

The project was approved by DUHREC (HEAG-191-2018). Participants who had attended a 3SOC performance and who had consented to be invited into the follow-up study during the post-performance survey (Study 1) were emailed invitations to complete a follow-up online survey in the months after their attendance. Personalised survey links were used to link Study 1 and Study 2 responses for respondents to Study 1. Additionally, organisations that had hosted a 3SOC performance were asked to forward an advertisement with an anonymous survey link to people who provided their email to the organisation when registering their attendance. Thus, audience members were able to respond to the follow-up survey regardless of whether they had responded to the post-performance survey.

Data collection for Study 2 commenced 1 month after the completion of Study 1 data collection. Given the time-period of data collection for Study 1 was more extensive than for Study 2 and data was pragmatically collected at multiple time points in both studies, it was not possible to tie the timing of Study 2 data collection to the timing of Study 1 data collection. Participants were therefore asked how long it had been since they saw the performance.

The survey took between 5 and10 min to complete. Reimbursement was provided to participants who provided a valid email address via entry into a draw to win one of six $30 Coles gift vouchers.

#### Data Analysis

Participant responses were analysed using the same analytic techniques as described in Study 1. This study included a more in-depth examination of the power of performance to produce lasting change in attitude and behaviour. This included direct comparisons of responses to Study 1 and 2 open-ended questions when these were able to be linked.

### Quantitative Results

Participants were asked how long it had been since they saw the 3SOC performance. Around a third (27%) had seen the performance less than six months ago, just over half had seen it 6–12 months ago (56.5%) and 16% had seen it over 12 months ago.

#### The Power of Performance

Table 4 displays the follow-up survey results for use of theatre or performance as a mechanism to connect and challenge assumptions about gambling. The data are broken down by community members and allied professionals. The findings revealed that almost all of the sample (99%) agreed that the use of theatre was an effective way to present the material and that performance by people who have experienced gambling harm was a powerful way of connecting to the issue.

**Table 4:** Follow-up survey results: the power of performance

Survey item	Community members	Allied professionals^a^	Combined sample
The use of theatre was an effective way to present the material	26 (100%)	63 (98.4%)	89 (98.9%)
Performance by people who have experienced gambling harm was a powerful way of connecting to the issue	26 (100%)	63 (98.4%)	89 (98.9%)

No statistical comparisons were conducted as 2 cells had < 5 participants; n = 90 (26 community members, 64 allied professionals); Proportion of sample indicating agree/strongly agree

^a^Excluding professionals in the gambling harm sector (gambling help professionals) (n = 17) and professionals who did not indicate the sector in which they were employed (n = 1)

#### General Knowledge and Attitudes About Gambling Harm

Table 5 displays the follow-up survey results for gambling knowledge and attitudes about gambling harm, broken down by community members and allied professionals. The majority of participants reported their understanding about gambling harm (92%) and empathy towards people with experiences of gambling harm (93%) had increased as a result of watching the 3SOC performance. At the time of the follow-up survey, the majority of participants agreed that they had a sound understanding of gambling harm (77%), understood why people may develop gambling harm (85%), understood the consequences of gambling harm (94%) and had empathy towards people who have experienced gambling harm (93%). There were no significant differences between the two subsamples on their responses to these items.

**Table 5:** Follow-up survey results: General knowledge and attitudes about gambling harm

Survey item	Community members	Allied professionals^a^	χ^2^	p-value	Combined sample
*Changes in general knowledge and attitudes about gambling harm*^*b*^					
Understanding about gambling harm	24 (92.3%)	57 (91.9%)	0.003	0.953	81 (92.0%)
Empathy towards people with experiences of gambling harm	25 (96.2%)	57 (91.9%)	0.513	0.474	82 (93.1%)
*Current general knowledge and attitudes about gambling harm*^*c*^					
Sound understanding of gambling harm	19 (73.1%)	49 (79.0%)	0.370	0.543	68 (77.2%)
Understanding about why people may develop gambling harm	24 (92.3%)	51 (82.3%)	1.469	0.225	75 (85.2%)
Understanding of the consequences of gambling harm	25 (96.2%)	58 (93.5%)	0.232	0.630	83 (94.3%)
Empathy towards people who have experienced gambling harm	23 (88.5%)	59 (95.2%)	1.294	0.255	82 (93.2%)

n = 88 (26 community members, 62 professionals)

^a^Excluding professionals in the gambling harm sector (n = 17) and professionals who did not indicate the sector in which they were employed (n = 1)

^b^Proportion of sample endorsing increased/strongly increased

^c^Proportion of sample indicating agree/strongly agree

#### Professional Attitudes and Practice

Table 6 displays the follow-up survey results from allied professionals regarding their professional attitudes and practice. The majority of allied professionals reported that their willingness to discuss gambling harm (84%), and confidence to ask clients about their gambling (83%), confidence in referring their clients for gambling treatment to appropriate services (83%) had increased as a result of watching the 3SOC performance. At the time of the follow-up survey, most allied professionals agreed that they were willing to discuss gambling harm with others (87%), but somewhat smaller proportions agreed that they were confident in asking clients about their gambling (71%) or referring their clients for gambling treatment to appropriate services (69%).

**Table 6:** Follow-up survey results: Professional attitudes and practice

Survey item	Allied professionals^a^
*Changes in professional attitudes and practice*^*b*^	
Willingness to in discuss gambling harm^c^	52 (83.9%)
Confidence in asking clients about their gambling^d^	38 (82.6%)
Confidence in referring clients for gambling treatment to appropriate services^d^	38 (82.6%)
Belief that it is important to identify gambling harm among clients attending non-gambling services^c^	52 (83.9%)
*Current professional attitudes and practice*^*g*^	
Willingness to discuss gambling harm with others^c^	54 (87.1%)
Confidence in asking clients about their gambling^f^	34 (70.8%)
Confidence in referring clients for gambling treatment to appropriate services^g^	34 (69.4%)
Importance of identifying gambling problems among clients attending non-gambling services^c^	61 (98.4%)
Importance of asking about gambling as part of organisation’s client intake form^f^	32 (66.7%)

Only administered to professionals

^a^Excluding professionals who did not indicate the sector in which they were employed (n = 1) or professionals in the gambling harm sector (n = 17)

^b^Proportion of sample endorsing increased/strongly increased

^c^n = 62

^d^n = 46;

^e^ Proportion of sample indicating agree/strongly agree

^f^n = 48

^g^n = 49

Most allied professionals (84%) reported that their belief that it is important to identify gambling harm among clients attending allied services (e.g., primary care, mental health, and AOD services) had increased as a result of watching the 3SOC performance. At the time of the follow-up survey, almost all allied professionals believed that it is important to identify gambling problems among clients attending allied services (98%), but only two-thirds believed that it is important to ask about gambling as part of their organisation’s client intake form (67%).

At the time of the follow-up survey, few allied professionals (n = 2, 5.0%) almost always informally asked their clients about their gambling, but approximately two-thirds indicated that they sometimes or often asked (n = 26, 65.0%) and just under one-third rarely or never asked (n = 12, 30.0%). Interestingly, over one-quarter indicated that their organisation has a gambling screen in its client intake tool (n = 11, 28.2%), but a larger proportion indicated that their organisation did not have a gambling screen in its client intake tool (n = 16, 41.0%); and a considerable minority (n = 12, 30.8%) did not know if their organisation had a gambling screen in its client intake tool.

### Qualitative Results

A total of 101 participants completed at least one of the open-ended items, the majority being allied professionals (n = 62) and community members (n = 24), with 15 gambling help professionals also responding. The power of performance to evoke lasting reflection and both attitude and behaviour change was an overarching theme to emerge from the data. It had two distinct elements: understanding gambling through lived experience and continued reflection sustaining change. These themes are explored below with a final section using linked findings from study 1 and 2 to evidence real shift from behaviour intention to actual behavioural change.

#### Understanding Gambling Harm through Lived Experience

The power of performance to bring the lived experience of gambling and associated harms to life for both gamblers and affected others was a strong theme across the data. The immersive experience continued to arouse intense emotional responses for many who still recalled specific moments or elements of the performance: “loud voices, chaos, silence and actors expressed feelings of hopelessness and despair” (gambling professional). The vast majority of participants demonstrated a deeper and more nuanced understanding of gambling, again commenting on the size of the problem, its causes, complexity and consequences, as well as reflections on the shame and embarrassment that is associated with gambling harms.

Some participants continued to feel shocked and even overwhelmed by the size and scope of the problem in Australia and the role of industry in this: “I didn’t realise how entrenched gambling was in the general community and how it affects the well-being of the partner and children” (gambling professional).

A common response from both professional and community audience members was to emphasise their deeper understanding of gamblers and reasons for gambling: “I have a much better understanding of the reasons people continue to gamble” (gambling help professional). Participants remembered key scenes demonstrating the underlying causes of gambling addiction, particularly how gambling represented an escape from loneliness, struggle and desperation: “The image of the housewife who had nowhere to go and found safety and companionship in a gambling venue as they were open all hours of the evening” (allied professional). They also continued to demonstrate an awareness of the complexity of the issue and the varying factors that can lead to, and sustain, a gambling addiction: “It has made me reflect on the individual’s journey and all the contributing factors that can lead to a gambling addiction” (gambling help professional).

The devastating consequences of gambling was a resonating theme for participants, with many talking about the impact and the toll gambling had taken on lives, either directly or indirectly: “Causes so much destruction to the person, their family members and those around them, but it’s possible to recover” (allied professional). Again, the power of hearing the lived experience directly from performers remained resonant with respondents recalling very specific scenes: “A performer speaking of the loss of her children” (community member).

Some recalled the shame and embarrassment of gambling problems and how this shame silenced the issue and prolonged its impact: “I think I've always realised the impact on the gamblers’ immediate family, the performance really enlightened [sic] the shame and worthless feelings of the gamblers themselves and the struggle they have. Like alcoholics not much cam[sic] be done until they own they have a problem” (allied professional).

These findings demonstrate how powerful an immersive performance can be, not only to elicit an immediate emotional response in audience members but to also lead to sustained reflection and deepening understanding over time. As discussed above, audience members showed a nuanced understanding of the causal factors associated with gambling addiction, as well as its complexity, consequences and harms.

#### Continued Reflection Sustaining Change

The power of the performance to invoke continued, deep reflection in the day-to-day lives of participants led to a sustained impact on their attitudes and behaviours, including in terms of *personal biases, feelings of compassion and empathy* as well as *changes in practice* among professionals.

Allied professionals explained how the performance humanised the experience for them: “Since the performance, the impact of gambling has been more humanised” (allied professional). This led to reflections on personal bias and changing attitudes.

“I was really moved by witnessing true stories told by the people who had experienced them - and learning about the wide variety of experiences which can lead to gambling harm definitely changes my attitude and called me to reflect on my own biases” (allied professional).

Importantly, both gambling and allied professionals articulated an increased level of sympathy and empathy towards those experiencing gambling harm, as they were now more aware of the individual and appreciative of the factors at play.

“I have a deeper understanding & empathy as often experiences for people are labelled as ‘gambling’. Yes, that is a behaviour but behind every behaviour is a hope & we need to be exploring the full experience of the individual to understand the trajectory of their journey. Often there are lots of layered experiences that lead people to gambling” (gambling help professional).

“We already have strong referral practices to gambling support within our program, however, my empathy and understanding of the emotional impact of these problems was heightened. I came away with a new sense of empathy for both the person experiencing gambling issues and their family.” (allied professional).

The more nuanced understanding of gambling and related harms led to changes in practice for many of those working in allied professions. This included being more comfortable asking about gambling: “easier to ask clients if they gamble” (allied professional); or simply being more alert to the presence of gambling issues. Some participants reported thinking more openly and exploring the issue in depth with clients as a result of having a deeper understanding of gambling, its onset and complex nature, and the way it feeds into other aspects of life: “I am more open to asking how people feel about gambling and how it might be affecting them or their family” (allied professional). Some mentioned how their treatment continues to become more person-centred, being more in tune with, and conscious of, the impact of gambling as a result of viewing the performance: “Always becoming more person-centred. Better knowledge of the connections between social issues and the impact on real people’s lives” (allied professional). One allied professional explained how they were now much more aware of gambling harm and more likely to recognise the underlying factors behind gambling.

“I am more likely to pick up on gambling being a possible source of comfort for people who are not in a good place, both in personal and professional realms; I am much more aware of gambling harm in general” (allied professional).

For those who did not work directly with people experiencing harm, practice change could be seen in terms of internal team discussions or changes to service provision or policy.

“I'm not currently working in direct client service provision - but I have included this issue in mentoring sessions that I've provided to junior staff, and I'm cognizant of some of these issues when talking with colleagues about service planning and delivery with a range of vulnerable clients (allied professional).

For others, practice change involved education and advocacy within the wider community.

“We now provide resources for gambling help, we purchased the movie/documentary “Ka Ching” and held a public screening and now have copies available to be borrowed by community members and organisations to increase awareness. Our toilets now have help information on the back of doors” (allied professional).

While most participants demonstrated clear evidence of sustained changes in attitude and/or behaviour regarding gambling harm, some participants reported little or no change. Most commonly this was because they felt they already had a clear understanding:”No, I have worked in the gaming industry for over 25 years and I know the impact gambling harm has on individuals and families” (gambling help professional); or, in the case of behaviour change, because they did not work directly with people experiencing harm from gambling.

### Comparing Responses over Time as Evidence of Change

Where participant data was collected at both time points, responses to open-ended questions about attitude and behaviour change were compared to explore whether audience members’ early expectations about personal attitude and behaviour change manifested over time. The pattern of responses showed good evidence that intentions articulated immediately post-performance did actualise into positive, sustained change over time. Table 7 provides example quotes evidencing where change occurred in line with participant’s intentions as well as examples of where the change went far beyond what the participant had anticipated. There were a few instances where comparisons of responses failed to reveal specific evidence of sustained behavioural change but only one participant overtly discussed a failure to implement expected practice change, demonstrating the importance of the work environment to support optimal professional practice.

**Table 7:** Comparison of individual participant responses to open-ended questions in Study 1 and Study 2

Participant type	Study 1 responses	Study 2 response
	*Quotes demonstrating attitudinal change*	
Allied professional	“I believe I’ll be much more empathetic and appreciative of people’s struggles”	“I have a much greater appreciation of the harm caused by gambling to individuals and families. I also appreciate the emotional struggle of addicts and their family members to a much greater level”
Allied professional	“More respectful.”	“I'm more empathetic to people affected by this addiction”
Allied professional	“I have probably held some negative beliefs about this—this performance was so moving. I feel I have definitely changed these beliefs”	“More empathy towards people with gambling issues”
Community member	“I’ve always been anti-gambling but now have more empathy and understanding”	“I have more empathy for those that may suffer gambling addiction and understand that a gambling problem is often a consequence of past or present traumatic experience”
Community member	“More empathetic. Less stigma. Less likely to assign individual blame”	“I also felt more empathetic towards people who experience gambling harm”
	*Quotes demonstrating behavioural change *^a^	
Allied professional	“More open to understanding/being sensitively curious to open a conversation”	“I have included this issue in mentoring sessions that I've provided to junior staff, and I'm cognizant of some of these issues when talking with colleagues about service planning and delivery with a range of vulnerable clients”
Allied professional	“Discuss more clients more [sic] and ask questions”	“It has helped me ask questions about gambling and identify the wide range of people gambling can affect”
Allied professional	“If I notice issues with money, I’ll be much more likely to ask if gambling is a problem for the person I’m talking to”	“I am much more likely to ask [callers to a crisis line] with financial difficulties if gambling is a problem for them or someone in their life. I am also much more confident in providing referrals where gambling is a problem”
Allied professional	“Being more open to ask about gambling. Being more in touch with the multi-faceted emotions associated with gambling… Ask the question”	“I ask more often about gambling, both to the client and about family members and their thoughts around this”
Allied professional	“Consider questions and invitations in regard to gambling and delicately pursue questioning”	“I have more compassion, and particularly changed how I engage people I suspect have gambling issues”
	*Quotes demonstrating change beyond initial expectations*	
Allied professional	“I will be far more aware of the harms of gambling” and “I will be far more empathetic when working with people who gamble”	“I feel even more compelled to do what I can to prevent gambling harm; I am more willing to speak up about gambling and try to educate others; I am now considering starting a project aimed at adolescents in schools, educating them about gambling, gambling harm, probability and chance and the false beliefs around gambling. I am now even more passionate about this subject and helping to change society's attitudes towards gambling.”
Allied professional	“I would ask direct questions about gambling”	“I screen for gambling as well as other addictions during every intake assessment”
	*Quote demonstrating a lack of change*	
Allied importance	“Definitely the importance of asking about gambling” and “I will ask young people about gambling”	“I found it really powerful and expected it would really stick with me but just in completing this questionnaire I have realized that my practice hasn't changed as much as I expected. I think this is likely due to lots of practice change happening organizationally and so it's hard to keep everything in mind”

^a^Question regarding practice change was only asked of professionals

## Discussion

This project (studies 1 and 2) aimed to explore whether exposure to a performance by Three Sides of the Coin (3SOC) was successful in achieving the group’s aim of increasing understanding regarding gambling harm and changing attitudes and behaviours of audience members, in particular allied professionals and community members. The combined data suggested that the performances were successful in increasing understanding and changing attitudes of audience members in the short term and of achieving the ultimate aim of sustained change to attitudes and practice in relation to gamblers and affected others in the longer term.

Prior research has shown that gamblers and affected others can experience stigma and shame due to gambling problems and harms and that this can result in a reluctance to seek support and treatment in a timely manner, potentially slowing recovery and increasing long gambling harms (Suurvali et al., 2009). However, many gamblers and affected others access other health services (Cowlishaw et al., 2013; Dowling et al., 2014, 2017) and talk to friends or family (Rodda et al., 2018), providing a range of opportunities for identification and early intervention. The key aims of 3SOC’s performances are to challenge understanding of gambling-related harm and, in turn, change attitudes and behaviours to ensure that gamblers and affected others receive empathy and support in their encounters with allied health, intersecting professions and the broader community.

Examination of the data collected immediately post-performance provided evidence that the performances were achieving the initial aim of increasing understanding, changing attitudes and behavioural intent of audience members in relation to gamblers and affected others. The quantitative data showed the vast majority of both allied professionals and community members thought that the performance was powerful and that it had increased their understanding about gambling harm and their empathy towards those affected by gambling harm. Importantly, professionals reported that their willingness and confidence to discuss gambling harm had also increased.

The qualitative data provided insight into these statistics. The deep immersive experience provided respondents with a far more nuanced understanding of gambling harm, including the size and scope of the problem in Australia and the complexity of the issue including its multi-layered causes and the wide ranging harms affecting both gamblers and those close to them. In particular, audience members recognised the level of stigma and shame associated with gambling. Participants described how this more nuanced understanding led them to feel more confident and willing to look for and ask about gambling issues. An important theme for allied professionals was an increased openness to explore gambling harm empathically within their practice in the future as a means of reducing shame and stigma and to support affected clients through their services and specialist referrals.

Data collected in the follow up study provided evidence of longer-term impact, with combined results showing that respondents maintained their understanding and continued to show more positive attitudes towards those affected by gambling harm, as well as demonstrating evidence of actual behavioural change. The quantitative data showed that the vast majority of participants continued to agree that their understanding about gambling harm had increased as a result of watching the 3SOC performance and that they now felt they had a sound understanding of gambling harm, including its causes and consequences, as well as feelings of empathy towards people who have experienced gambling harm.

A closer examination of the attitudes and behaviours of allied professionals within the quantitative data showed that almost all professionals believed it was important to identify gambling problems amongst their clientele and most felt that this belief had increased as a result of watching the 3SOC performance. The vast majority also reported increased confidence to ask clients about their gambling and to refer clients to appropriate services as a result of watching the 3SOC performance. In terms of change within their own practice, about two-thirds of allied professionals believed that it was important to ask about gambling systematically as part of their organisation’s client intake process. Further, most allied professionals said they now felt confident in asking clients about their gambling and providing referrals, with a similar proportion reporting asking their clients about their gambling at least some of the time. These findings suggest that this performance overcame some of the barriers to screening in allied clinical service settings by increasing the confidence and capability of allied health professionals to feel confident to identify gambling harms in their clients (Manning et al., 2020; Rodda et al., 2018). However, only one-quarter of allied professionals indicated that their organisation has a gambling screen in its client intake tool, with many indicating that there was no such screen in their organisation intake tool or they did not know whether there was such as a screen in their organisation intake tool. These findings suggest that while there were potentially changes to individual clinician attitudes and practices, this had not necessarily extended to service-wide practices.

The qualitative data again provided important insight and nuance to findings from the quantitative data showing that theatre was a powerful way to connect to the issue. The data demonstrated that the deep immersion of the performance kept the lived experience alive, with participants continuing to recall specific scenes such as the shame and embarrassment felt by the former gamblers and affected others, discussions about the causes of gambling problems and the high burden of harm. This resulted in continued reflection and a deepening of understanding regarding the complexity of gambling for participants. Continued reflection then led to sustained change in terms of participants’ attitudes and behaviours. This included reflecting on personal biases and increased feelings of compassion and empathy towards those affected by gambling. Importantly, the findings provided more evidence of practice change, with professionals discussing an increased comfort and confidence in asking about gambling and being alert to the presence of gambling issues. Some discussed being more open to exploring gambling with clients within their practice.

Together these findings provide evidence that 3SOC storytellers’ use of artistic methods to share personal stories in a powerful, emotive way with audiences as a means of disruption and eliciting personal critical reflection is successful. The results also support prior research finding that performance arts can be a valuable means of increasing understanding, challenging attitudes, and changing behaviour both within health professionals and in the broader community (Crisp & Taket, 2022; Fraser & Sayah, 2011; Quek et al., 2012).

Some professionals, however, reported little or no change to their practice. The qualitative data provided important insight in relation to this issue, with participants explaining this was because they already had a deep understanding or because they did not work directly with clients affected by gambling. This latter finding may, at least to some extent, explain the earlier quantitative finding that a substantial proportion of respondents said they rarely or never asked about gambling and were unaware whether their organisation screened for gambling issues. Interestingly, some of those who did not work directly with gamblers or affected others still found opportunities to influence change within their workplaces, such as through organisational professional development, changes to policy or broader community education and advocacy.

Importantly, the methodology used in this project facilitated an examination of individual responses across time to see if, when and how early intentions with regard to attitudes and behaviours actualised into change over time. There was evidence of sustained change in line with intentions including showing increased empathy and respect towards those affected by gambling, initiating open conversations about gambling as well as efforts to identify gambling issues and respond appropriately. The interrogation of data across time also identified an instance where a health professional with good intentions was unable to implement desired practice change due to organisational pressures, demonstrating the importance of organisational support to facilitate optimal professional practice (Manning et al., 2020; Rodda et al., 2018).

### Strengths and Limitations

A strength of this research project was the integration of people with lived experience of gambling harm throughout the project, informing on research design as well as the interpretation and write-up of findings. Another strength of the project was the examination of both short and longer-term change through collecting data at two time points. This provided evidence of sustained attitudinal change and change in professional practice. The study would have been further strengthened by including a longer timeframe for follow up or an additional follow up to examine long-term change. A limitation of the study was that no data was collected prior to the performance. A pre-post study design would have allowed more rigorous evidence to be gathered in terms of change in understanding, attitudes, and behaviour as a result of the performance.

### Conclusion

In conclusion, this evaluation showed that 3SOC performances were successful in their aim of increasing understanding regarding gambling in its audiences, as well as changing attitudes and behaviours. The post-performance survey showed clear evidence of increased and more nuanced understanding of gambling in both allied professionals and community audience members. In addition, there was evidence of positive shifts in attitude and behavioural intention, including increased empathy and a willingness to discuss gambling harm and support those affected. Importantly, the follow-up survey demonstrated that these positive changes persisted over time, with participants demonstrating a nuanced understanding of gambling and increased empathy towards those affected. There was also evidence of practice change in allied professionals, who now felt confident to ask about gambling and provide appropriate support. One of the reasons for this sustained impact over time was the use of performance based on lived experience as a professional education tool, with findings showing that being fully immersed in the lived experience of gamblers and affected others through live performance was a powerful way of connecting to the issue at both an emotive and cognitive level. This then resulted in continued reflection by audience members leading to a deep, nuanced understanding of gambling and sustained attitudinal and behavioural change.

## Data Availability

The data that support the findings of this study are available from the corresponding author upon reasonable request.
